# Vestibular signal processing in a subject with somatosensory deafferentation: The case of sitting posture

**DOI:** 10.1186/1471-2377-7-25

**Published:** 2007-08-29

**Authors:** Jean Blouin, Normand Teasdale, Laurence Mouchnino

**Affiliations:** 1 Laboratoire de Neurobiologie de la Cognition, CNRS and Aix Marseille Université, 3 Place Victor Hugo, 13331 Marseille, France; 2 Faculté de Médecine, Division de kinésiologie, Université Laval, Québec, Canada; 3 Centre de recherche du CHA et Centre d'excellence sur le vieillissement, Hôpital Saint-Sacrement, Québec

## Abstract

**Background:**

The vestibular system of the inner ear provides information about head translation/rotation in space and about the orientation of the head with respect to the gravitoinertial vector. It also largely contributes to the control of posture through vestibulospinal pathways. Testing an individual severely deprived of somatosensory information below the nose, we investigated if equilibrium can be maintained while seated on the sole basis of this information.

**Results:**

Although she was unstable, the deafferented subject (DS) was able to remain seated with the eyes closed in the absence of feet, arm and back supports. However, with the head unconsciously rotated towards the left or right shoulder, the DS's instability markedly increased. Small electrical stimulations of the vestibular apparatus produced large body tilts in the DS contrary to control subjects who did not show clear postural responses to the stimulations.

**Conclusion:**

The results of the present experiment show that in the lack of vision and somatosensory information, vestibular signal processing allows the maintenance of an active sitting posture (i.e. without back or side rests). When head orientation changes with respect to the trunk, in the absence of vision, the lack of cervical information prevents the transformation of the head-centered vestibular information into a trunk-centered frame of reference of body motion. For the normal subjects, this latter frame of reference enables proper postural adjustments through vestibular signal processing, irrespectively of the orientation of the head with respect to the trunk.

## Background

The control of human upright and seated postures is based on information about body orientation and motion [1,2]. Neck-muscle proprioception plays a crucial role to this process as it allows the central nervous system to create an internal estimate of body motion through visual and vestibular signals [3-7].

Supporting this view is the early seminal observation made by De Jong and colleagues [8] and Cohen [9] of extensive sensorimotor deficits resulting from injection of local anaesthetics in the neck in animals as well as in humans [8], or from sections of the dorsal roots in monkeys [9]. For instance, in humans, the interruption of afferent flow from neck muscles induces ataxia, staggering gait, hypotonia of lower and upper segments, and a sensation of falling [8].

Evidence for cervical and vestibular neural integration is found in the rostral fastigial nuclei. In these deep cerebellar nuclei, on which massive vestibular and somatosensory signals converge, body-in-space motions are represented in a body frame of reference rather than in a head-based frame of reference [5,10,11]. Testing a subject with intact afferent information of the cervical region but deprived of proprioception below the neck, Day and Cole [12] showed that, together, the cervical and vestibular signals could provide the required estimate of body-in-space orientation/motion to keep equilibrium when seated. Interestingly, this case report showed the possibility to control posture in the absence of contact information (e.g. cutaneous and pressure) of the body with the supporting surface. As discussed above, neck proprioception may have allowed this subject determination of body motion through vestibular signals even in the absence of proprioception of trunk and limbs muscles.

Here we investigated if, alone, the vestibular signals, which contribute to the perception of head motion/orientation in space and which have connections with motoneurons of axial and proximal postural muscles, are sufficient to control sitting posture. This was done testing the capacity of a rare subject, with a large-fiber sensory neuropathy that resulted in a severe loss of position sense below the nose (including the cervical region), to maintain a sitting posture. The contribution of vestibular information was specifically tested using two methods. One method consisted in creating a subliminal mismatch between vestibular information and actual body motion by slowly rotating the DS's head towards either shoulder in the dark. Controlling balance through vestibular information after undetected change of head-trunk configuration should lead to increase instability as the vestibular signals will no longer inform about the veridical body-in-space displacements.

The second method employed in the present experiment consisted in externally stimulating the labyrinthine apparatus (galvanic vestibular stimulation technique, GVS). GVS produces a pattern of irregularly firing vestibular afferents that resembles that of the natural response to linear or angular head acceleration [13,14]. When applied to unrestrained subjects, GVS induces body tilt toward the anode side [7,15,16]. Day and Cole [12] showed that in a subject without body proprioception but intact cervical afferent signals, GVS also induced body tilt towards the anode side, but with a much greater magnitude than in control subjects. Here we tested if similar responses to GVS would be observed when neck muscle proprioception is also severely impaired.

## Methods

### Case report

The deafferented subject (female, 55 years-old) suffered at the age of 31 from a loss of the large myelinated fibres from her whole body after a severe sensory polyneuropathy. Neurophysiological data of the DS have been reported elsewhere [17]. In summary, at 27 years old, the subject suffered from a first episode of acute polyneuropathy with a complete paralysis including the respiratory muscles. A diagnostic of Guillain-Barré was made. It took six months for the subject to completely recover from the syndrome. A second episode of extensive sensory polyneuropathy occurred suddenly four years later which affected selectively the large myelinated sensory fibers. At this time, and since then, the DS has a severe loss of all somatosensory modalities (kinaesthesia, tendon reflexes, touch, vibration, pressure) below the nose. The vestibular nerves remained intact as confirmed by assessment of her vestibulo-ocular reflex [18]. Electrophysiological investigations showed no evidence of motor fibers impairment but the DS cannot stand upright or walk. Studies have shown that the DS can scale force production with similar accuracy as control subjects [19,20] and that under visual control, she can perform accurate reaching, grasping and weight judging tasks [21-25]. Since her deafferentation, the DS has never attempted to seat without back support, as requested in the present study. Prior to the experiment, she was uncertain if she could do so without visual feedback. Despite the control of seated posture in healthy subjects has been described in details elsewhere [26-28], three control subjects also participated to the experiment for comparison (mean age: 54 years).

### Experimental setup

The experiment was carried out in a dim room such that the subjects could well see their trunk, legs and the experimental room with the eyes open but could not perceive change of luminance with the eyes closed when the experimenter rotated their head (for the DS, a change of luminance could have informed her about head rotations). The subjects sat on an 60 × 120 cm AMTI force platform placed on a 70 cm high plinth. No back or arm support was provided. Both legs were dangling with the hands resting on the subjects' thighs. Small spherical retroreflective markers were placed on both infraorbital margins, sternum, and at the center of the forehead. They allowed recording movements of the head using an automatic TV-image processor (E.L.I.T.E. system) at 100 hz. The center of pressure (CoP) exerted on the platform was recorded at 500 Hz. Subjects wore a light helmet on top of which was fixed a laser diode. This laser beam helped the experimenter to change the orientation of the subjects' head with respect to their trunk as will be specified below.

### Experimental conditions

All subjects were tested in three main conditions:

#### 1) Head centered

The head was centered on the trunk. This condition was performed with the eyes closed or open.

#### 2) Head rotated

This condition was also performed with the eyes closed or open. For the trials with the eyes closed, prior to data acquisition, an experimenter rotated the subject's head very slowly in the horizontal plane (in about 15 s) until the laser beam centered on the head hit a 5 m distant target located 55° on either side of the subject. At the end of the trial, the DS kept the eyes closed and an experimenter grasped her shoulders while another brought her head back to the primary, central position rotating it back and forth a few times (3–4 rotations) at moderate speed (i.e. clearly perceptible by the DS). This procedure allowed the DS to re-open the eyes rapidly after the trials without having her realizing that her head had been previously rotated. The DS was simply told that these 3–4 passive head rotations were part of the experiment. To preserve homogeneity between conditions, similar procedures were taken for the Head centered condition (without vision). For the trials performed with the eyes open, the experimenter also slowly rotated the subject's head (in about 5 s here). With the eyes closed, the DS never reported perception of the slow (i.e. 15 s) head rotations introduced before the recordings. After the experimental session, when asked about the sensation she had during the experiment, the DS clearly expressed that she never perceived change of head orientation that occurred prior to data acquisition when she had the eyes closed. This confirms that, contrary to the patient tested by Day and Cole [12], the DS tested here showed a severe lost of somatosensory information at the cervical region.

#### 3) Galvanic vestibular stimulation (GVS)

This condition was only performed with the eyes closed. The subject had the head centered on the trunk. Two seconds after the onset of recording, a rectangular, bipolar binaural direct electric current was delivered via 3 cm electrodes taped over the mastoid processes of the subject. The stimulations lasted 5 s and their magnitudes were either 0.75 or 1.5 mA. GVS produces a pattern of irregularly firing vestibular afferents that resembles that of the natural response to linear or angular head acceleration [13,14]. When applied to unrestrained subjects, GVS induces body tilt toward the anode side [7,15,16,29]. The purpose for the GVS-evoked body tilt is still uncertain. Recent studies suggest that it could be an automated response delivered by the postural control system to stabilize the head in gravito-inertial space [29].

The trials lasted 10 s in all conditions. The Head centered and Head rotated conditions were first performed with the eyes closed and then repeated with the eyes open (the laser diode fixed on the head was always extinguished during the recordings with the eyes open). Each subject performed eight trials per head orientation (randomly presented) for both viewing conditions (total of 48 trials). The GVS condition was ran after the Head centered and Head rotated conditions. Eight repetitions, randomly presented, were given by subject per type of stimulation (total of 32 trials). Rests of about 45 s were given after each trial during which the DS could lean against a portable back support put in place by the experimenter. The experiment was approved by the local ethics committee and was carried out in compliance with the Helsinki Declaration.

## Results

**Figure 1:**
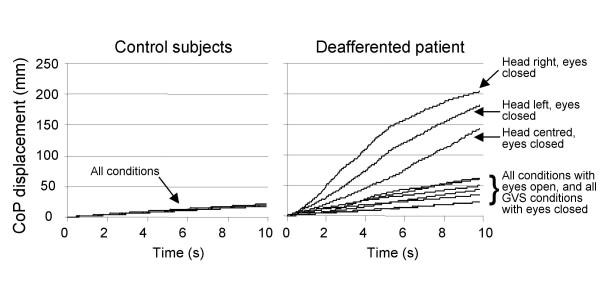
**Length of CoP displacements**. The graphs show the mean CoP displacement over time in the different experimental conditions for the control subjects (left panel) and the deafferented subject (right panel). As the control subjects were remarkably stable while seated in all experimental conditions, the different traces are superimposed. The deafferented subject maintained a relatively stable seated posture with the eyes open. Closing the eyes markedly deteriorated her stability, especially when she had the head unconsciously turned towards a shoulder. Electric stimulation of the vestibular apparatus when the deafferented subject had the eyes closed allowed her to roughly recover the body stability she had with the eyes open. See Additional file 1: Table 1 for the statistical analyses.

For each trial, the length of CoP displacement, as measured over the 10 s recording periods, was used as an index of postural stability. The results obtained by the DS for each experimental condition are given in the additional tables (Table 1 and 2) which are too extensive to be presented herein. With the eyes open, the DS showed a stable posture as demonstrated by her CoP displacement (average = 27 mm, SD = 6 mm) that was only slightly greater than that of the controls (average = 19 mm, SD = 1 mm) (Fig. 1, additional file 1: Table 1). Varying the position of the head on the trunk had no significant effect on the DS's postural stability when vision was available.

Dynamic representations of the DS's CoP displacements for representative single trials of the Head centered and Head rotated eyes closed conditions are shown in the additional file 3: Movie1. The DS's CoP displacement markedly increased with the eyes closed. On average, this increase was more pronounced when the DS had the head unconsciously turned towards the left (181 mm, SD = 43 mm) or the right (204 mm, SD = 40 mm) shoulder than when the head was centered on the trunk (144 mm, SD = 51 mm) (Fig. 1). The direction of the CoP displacement varied both within and between trials but did not depend of the head orientation. The sitting posture of the control subjects was remarkably stable. Withdrawing vision or turning the head had no significant effect on their CoP displacement (average = 19 mm, SD = 1 mm).

Turning the head toward a shoulder produces large muscular stretch. For the DS, in the absence of vision, because of the visco-elastic properties of the muscles, this muscular stretching resulted in passive movements of the head towards the neutral position. On average, after the 10 s recordings, the head was still deviated on the trunk by 38.7° and 21.2° in the rightward and leftward head rotations, respectively. The magnitude of the return movements of the head was therefore larger when the head was rotated towards the left than when it was rotated towards the right. The fact that magnitude of the CoP excursion of the DS was smaller when the head was rotated to the left, suggests no or negligible effects of these passive head movements on the CoP.

**Figure 2:**
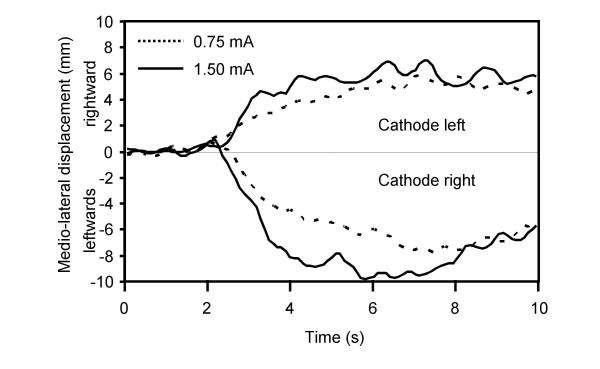
**Medio-lateral CoP displacements in GVS trials**. The graph show the mean medio-lateral CoP displacement of the deafferented subject recorded in trials with 0.75 mA or 1.5 mA galvanic vestibular stimulations. The GVS produced large lateral shifts of the CoP towards the anode side. The data from the healthy control subjects are not represented as the stimulations were not large enough to induce clear postural responses when seated. See additional file 2: Table 2 for the statistical analyses.

The primary postural responses to GVS were markedly larger in the DS than in the controls who did not show clear responses to the stimulations. The responses consisted in a shift of the DS's CoP essentially oriented towards the anode side (Fig. 2, additional file 2: Table 2; additional file 4: Movie 2). For the DS, the magnitude of the maximal CoP lateral displacements tended to increase with the magnitude of the stimulation (means of 7.4 mm SD = 3.7 mm and 10.2 mm SD = 3.5 mm for the 0.75 and 1.5 mA, respectively). The DS reported that GVS produced small cutaneous sensations behind the ears but did not report body tilt sensation. Before and after the GVS-evoked lateral shift, the DS was remarkably stable. This was unexpected given the large body oscillations showed by the DS in the first conditions performed with the eyes closed (including when her head was centered on the trunk). Indeed, the total CoP excursions measured over the 10-s periods were much smaller with GVS (means of 46 mm and 62 mm, for the 0.75 mA and 1.5 mA stimulations, respectively) than without GVS (144 mm). The great stability of the DS in conditions with GVS can be seen during the first 2 s and the last 3 s of the recordings shown in Fig. 1 and Fig. 2, and in the additional file 4: Movie 2 during which no GVS stimulation was delivered. Moreover, the CoP did not return towards its primary position when GVS stopped. This phenomenon, which has been frequently reported, is usually interpreted as a sign that at the offset of the stimulation, movement related signal evoked by GVS stopped rather than reversed [12,15].

## Discussion

Here, we show the remarkable capacity of a subject with a severe somatosensory loss (including cervical information and cutaneous cues with the sitting platform) to maintain an active sitting posture (i.e. without back or side rests) in the absence of vision. We argue that the DS controlled her posture through vestibular signal processing. Compelling evidence for a vestibular-based postural control comes from the marked increase of postural oscillations when the DS's head was unconsciously deviated from its primary, visually perceived, straight-ahead position. In this situation, and in the lack of head-on-trunk position information, the vestibular signals did not provide the DS with veridical information about her trunk displacements in space. Processing vestibular signals with such undetected head-trunk configuration is likely to have led to inappropriate postural adjustments with respect to the actual body oscillations. For instance, with the head unconsciously rotated to the left, the DS most likely detected (either consciously or unconsciously) backward displacements when she was actually moving leftward. Such craniotopic updating of body displacement with neck proprioception deficit presumably resulted in series of inappropriate postural adjustments in response to the vestibular stimulations.

A great deal of the evidence for the contribution of vestibular signals to the control of posture comes from studies conducted with labyrinthine-defective subjects [e.g. [30,31]] and from studies that tested the effects of external labyrinthine stimulations on healthy subjects (e.g. galvanic [7,15,16] or caloric [32] stimulations). While these studies indisputably attest the importance of vestibular information in postural control, the activation of the vestibular system resulting from such stimulations (or the lack of activation in the case of the patients) does not correspond to that normally arising from the tested postural tasks. In the present experiment, creating a mismatch between body motion and vestibular information by rotating the DS's head subliminally, proved to be an efficient way to demonstrate that the vestibular afferent signals generated by the body oscillations while seated were also contributive. Control subjects, for whom cervical afferent signals provide reliable information about head-trunk configuration, could transform the primary head-centered vestibular information into a trunk-centered frame of reference of body motion with the eyes closed [5,10,11].  Consequently, their balance was not perturbed when their head was rotated toward a shoulder. Interestingly, the difference in body stability between the DS and controls almost completely vanished when vision was available. This was true whether or not the head was rotated. With vision, the DS could presumably refresh her body image according to the new, visually-perceived, orientation of the head relative to the trunk. This would allow her to estimate body motion through visuo-vestibular integration and to produce postural adjustments accordingly. With or without visual feedback, it is most likely that the control of posture required a great deal of attention for the DS [33].

Despite her severe loss of neck muscle proprioception, the DS's postural responses to GVS were still mainly oriented towards the anode side. For safety reason and in order to preserve the confidence of the DS regarding the experimental procedures, we did not deliver GVS when the DS had the head unconsciously deviated towards a shoulder. The large lateral CoP shifts of the DS following GVS support previous findings obtained in individuals with somatosensory deficits [12,34]. GVS had no or only marginal, and relatively insignificant, effect on the controls' CoP. This result also confirmed other studies that have employed GVS in sitting conditions [12,35]. For control subjects, proprioception provided massive flow of information related to their actual body configuration and balance. Greater stimulations may be necessary to evoke larger postural responses, especially in conditions characterized by large surface of support, as in the present experiment.

Both the large CoP displacement observed when the DS had the head unconsciously deviated on the trunk and the large CoP shift after GVS onset suggest great sensitivity of the DS to vestibular stimulations. Such increased sensitivity has been reported when proprioceptive sense is deteriorated [12,18,30] or when body stability is unsecured [30,35,36]. Increased influence of vestibular information for the DS could result from the absence of the gating effect of proprioception on the vestibulospinal drive which is usually observed in control subjects [38,39]. Also, response to vestibular stimulation may have been augmented by the high level of background muscular activity [40,41] presumably present in the DS while seated without visual information.

An unexpected result of the present experiment was the DS's increased body stability for the GVS condition as compared to the condition without GVS (Head centered condition, eyes closed). Indeed, before and after the GVS-evoked large shift towards the anode side, the DS's CoP remained relatively stationary. Reasons for this improved balance in the GVS session remain uncertain. This experiment was the first attempt of the DS to sit without back or side supports since her deafferentation. It is then plausible that visual information, which was available in the conditions ran prior to the GVS condition (i.e. Head centered and Head rotated conditions with eyes open), allowed the DS to acquire a new strategy to control her sitting posture. This strategy may involve stiffening and freezing the trunk and legs, a strategy used by patients and elderly individuals with sensory impairments to control their balance while standing and walking [42-44]. Of course, such a strategy would certainly prove to be inefficient for contexts where the posture would be perturbed externally [45]. The lack of muscular activity recordings in the present experiment prevents verification of this hypothesis.

## Conclusion

Altogether, this case report demonstrates that, in the case of a severe loss of proprioception of the body and of the cervical region, and contact information with the surface of support, seated posture can be maintained without vision through vestibular signal processing. However, the optimization of this vestibular-based control of posture requires an accurate representation of the head orientation on the trunk.

## Competing interests

The author(s) declare that they have no competing interests.

## Authors' contributions

JB and LM conceived the study and collected the data. All authors evaluated the data, performed data analyses and wrote the manuscript. All authors read and approved the final manuscript.

## Pre-publication history

The pre-publication history for this paper can be accessed here:



## Supplementary Material

Additional file 1 **Table 1**. T-tests comparing the length of the CoP displacements of the deafferented subject in all experimental conditions. Click here for file

Additional file 3 **Movie 1**. Dynamic representations of the deafferented subject's CoP displacements for representative single trials of the Head centered and Head rotated eyes closed conditions. Click here for file

Additional file 2 **Table 2**. T-tests comparing the maximal CoP lateral shifts produced by the deafferented subject in the GVS conditions. Click here for file

Additional file 4 **Movie 2**. Dynamic representations of the deafferented subject's CoP displacements for representative single trials of the GVS conditions. Click here for file
