# Supplementary material for: Vestibular signal processing in a subject with somatosensory deafferentation: The case of sitting posture
Source: BMC Neurol. 2007 Aug 29;7:25. doi: 10.1186/1471-2377-7-25 (PMC2014758; doi:10.1186/1471-2377-7-25)
Supplement: Additional file 1 — Table 1. T-tests comparing the length of the CoP displacements of the deafferented subject in all experimental conditions. [file 1471-2377-7-25-S1.pdf]

Table 1. Results of the *T*-tests comparing the length of the CoP displacement of the deafferented patient in the different sitting conditions

	Head centred Eyes closed (144mm**)	Head centred Eyes open (23 mm)	Head right Eyes closed (204 mm**)	Head right Eyes open (23 mm)	Head left Eyes closed (181 mm**)	Head left Eyes open (34 mm)	GVS 0.75 mA (43 mm**)	GVS 1.5 mA (61 mm**)	GVS -0.75 mA (49 mm**)	GVS -1.5 mA (63 mm**)
Head centred Eyes closed (144mm**)	-------	p<0.0001	p<0.01	p<0.0001	p>0.05*	p<0.0001	p<0.0001	p<0.0005	p<0.0005	p<0.001
Head centred Eyes open (23 mm)	p<0.0001	-------	p<0.0001	p>0.05	p<0.0001	p<0.0001	p<0.0001	p<0.0001	p<0.01	p<0.0001
Head right Eyes closed (204 mm**)	p<0.01	p<0.0001	-------	p<0.0001	p>0.05	p<0.0001	p<0.0001	p<0.0001	p<0.0001	p<0.0001
Head right Eyes open (23 mm)	p<0.0001	p>0.05	p<0.0001	-------	p<0.0001	p<0.0001	p<0.0001	p<0.0001	p<0.01	p<0.0001
Head left Eyes closed (181 mm**)	p>0.05*	p<0.0001	p>0.05	p<0.0001	-------	p<0.0001	p<0.0001	p<0.0001	p<0.0001	p<0.0001
Head left Eyes open (34 mm**)	p<0.0001	p<0.0001	p<0.0001	p<0.0001	p<0.0001	-------	p>0.05	p<0.0005	p>0.05	p<0.001
GVS 0.75 mA (43 mm**)	p<0.0001	p<0.0001	p<0.0001	p<0.0001	p<0.0001	p>0.05	-------	p<0.05	p>0.05	p<0.01
GVS 1.5 mA (61 mm**)	p<0.0005	p<0.0001	p<0.0001	p<0.0001	p<0.0001	p<0.0005	p<0.05	-------	p>0.05	p>0.05
GVS -0.75 mA (49 mm**)	p<0.0005	p<0.01	p<0.0001	p<0.01	p<0.0001	p>0.05	p>0.05	p>0.05	-------	p>0.05
GVS -1.5 mA (63 mm**)	p<0.001	p<0.0001	p<0.0001	p<0.0001	p<0.0001	p<0.001	p<0.01	p>0.05	p>0.05	-------

For GVS, negative mA is given when the cathode was on the left. *The mean obtained in the "Head left eyes closed" condition fell outside the 94% confidence interval computed in the "Head centred eyes closed" condition. ** The mean fell outside the 95% confidence interval obtained by the control subjects.
