# Supplementary material for: Vestibular signal processing in a subject with somatosensory deafferentation: The case of sitting posture
Source: BMC Neurol. 2007 Aug 29;7:25. doi: 10.1186/1471-2377-7-25 (PMC2014758; doi:10.1186/1471-2377-7-25)
Supplement: Additional file 2 — Table 2. T-tests comparing the maximal CoP lateral shifts produced by the deafferented subject in the GVS conditions. [file 1471-2377-7-25-S2.pdf]

Table 2. *T*-tests comparing the maximal CoP lateral shift produced by the deafferented patient in the GVS conditions

	Cathode left		Cathode right	
	0,75 mA (6.80 mm)	1,5 mA (9.06 mm)	0.75 mA (-7.98 mm)	1.5 mA (-11.35 mm)
Cathode left				
0,75 mA (6.80 mm)	-------	p>0.05*	p<0.0001	p<0.0001
1.5 mA (9.06 mm)	p>0.05*	-------	p<0.0001	p<0.0001
Cathode right				
0.75 mA (-7.98 mm)	p<0.0001	p<0.0001	-------	p>0.05**
1.5 mA (-11.35 mm)	p<0.0001	p<0.0001	p>0.05**	-------

Negative signs represent leftward shifts of the CoP. * The mean obtained in the "1.5 mA cathode left" condition fell outside the 94% confidence interval computed in the "0.75 mA cathode left" condition. **The mean obtained in the "1.5 mA cathode right" condition fell outside the 92% confidence interval computed in the "0.75 mA cathode right" condition.
